# A Meta-Analysis on the Association Between *TNFSF4* Polymorphisms (rs3861950 T > C and rs1234313 A > G) and Susceptibility to Coronary Artery Disease

**DOI:** 10.3389/fphys.2020.539288

**Published:** 2020-11-26

**Authors:** Shuyan Liu, Xiju Wang, Shoujun Yu, Miao Yan, Yue Peng, Guilong Zhang, Zhaowei Xu

**Affiliations:** ^1^School of Pharmacy, Binzhou Medical University, Yantai, China; ^2^Yantai Affiliated Hospital of Binzhou Medical University, Yantai, China

**Keywords:** polymorphism, coronary artery disease, meta-analysis, susceptibility, TNFSF4

## Abstract

**Background:** Coronary artery disease (CAD) remains the leading cause of mortality worldwide, and its susceptibility is closely associated with genetic modifications. The association between inflammation and CAD has been investigated in detail. This meta-analysis was conducted based on the PRISMA guidelines to evaluate the association between the *tumor necrosis factor superfamily member 4 (TNFSF4)* gene polymorphisms (rs3861950 T > C and rs1234313 A > G) and the risk of CAD.

**Methods:** The selected criteria included 11 eligible articles containing 18 studies (nine studies included 7,395 cases and 5,296 controls for rs3861950 and nine studies with 6,951 cases and 4,959 controls for rs1234313). Correlations between the two polymorphisms and CAD were estimated by pooling the odds ratios (ORs) with 95% confidence interval (95% CI) in allelic, dominant, recessive, heterozygous, and homozygous models.

**Results:** The pooled analyses demonstrated that the rs3861950 T > C polymorphism was significantly associated with an increased risk of CAD in the Asian population in the allelic model, dominant model, and homozygous model. Furthermore, subgroup analysis based on disease type showed that *TNFSF4* rs3861950 T > C had a robust correlation with increased risk of cerebral infarction (CI) in the allelic model, dominant model, heterozygous model, and homozygous model. However, the rs1234313 A > G polymorphism mostly tended to decrease the risk of CAD in the Asian and Caucasian populations in the allelic and dominant model. This single nucleotide polymorphism (SNP) had a close relation to myocardial infarction (MI) susceptibility in the allelic model, dominant model, and heterozygous model.

**Conclusion:** This meta-analysis identified two novel SNPs in *TNFSF4* significantly associated with CAD susceptibility.

## Introduction

Coronary artery disease (CAD) is a severe condition caused by the accumulation of plaque in the coronary arteries that leads to dysfunction of coronary arteries endothelial cells at an early stage (Marenberg et al., 1995; Kessler et al., 2016). Epidemiological and translational medical research has improved our understanding the mechanism underlying CAD (Khera and Kathiresan, 2017). However, CAD remains the leading cause of mortality worldwide. An increasing number of studies demonstrated that age, race, gender, smoking status, blood pressure, diabetes, obesity, and an unhealthy lifestyle are associated with an increased risk of CAD (Kessler et al., 2016). High-throughput sequencing and functional studies have confirmed that the susceptibility of CAD is regulated by a combination of genetic and environmental factors. Moreover, family-based studies indicated that the heritable variation was closely associated with CAD susceptibility (Marenberg et al., 1995). Therefore, there is a current need to identify robust genetic markers for the precise diagnosis of CAD in a specific population.

The close association between atherosclerosis and inflammation is well-known as the pathological basis of CAD(Christodoulidis et al., 2014). Furthermore, many well-established inflammatory mediators were thought to be potential clinical biomarkers of CAD. For example, TNFSF4, also called OX40L, belongs to the tumor necrosis factor superfamily and it is a membrane-bound protein predominantly expressed on antigen-presenting cells, CD4/8-positive T cells, and vascular endothelial cells (Baum et al., 1994). TNFSF4 has been suggested to bind to its receptor TNFRSF4 and improves the proliferation and development of memory T cells (Van Wanrooij et al., 2007; Kaur and Brightling, 2012). Previous studies have found that a high expression of TNFSF4 may play a vital role in atherosclerotic lesions and the accumulation of plagues in the arteries (Van Wanrooij et al., 2007). Therefore, there is a current need to improve our understand of the association between *TNFSF4* variations and CAD risks. Single nucleotide polymorphisms (SNPs) are associated with disease susceptibility by altering gene expression or function. Human *TNFSF4* gene maps to chromosome 1q25 and many SNPs have been found in the *TNFSF4* gene (Baum et al., 1994). Furthermore, case-control studies have already shown a robust association between some *TNFSF4* SNPs and CAD, such as rs17568, rs3861950, rs1234314, rs1234313, and rs3850641(Wang et al., 2019). Recently, a meta-analysis has reported the SNPs of *TNFSF4* and evaluated the associations between several SNPs and CAD risk(Wang et al., 2019). However, conclusions were drawn from insufficient data, and this caught our attention. Considering that no significant association was found between *TNFSF4* rs3861950 T > C and rs1234313 A > G polymorphisms and CAD ris, there is a need to re-evaluate the potential risks of CAD based on additional case-control studies.

## Methods

### Protocol and Registration

The implementation of this meta-analysis was completed in accordance with the “Preferred Reporting Items for Systematic Reviews and Meta-Analyses” (PRISMA) guidelines (Moher et al., 2009). The PRISMA checklist is provided in Supplementary Table 1.

### Literature Search Strategy

A systematic and comprehensive literature search based on the Participant, Intervention, Comparison, Outcomes, and Studies (PICOS) framework was performed in PubMed, EBSCO, Web of Science, and CNKI databases to collect the relevant articles published prior to February 2020 without language restrictions using the following terms and keywords: (tumor necrosis factor superfamily member 4” OR “TNFSF4” OR “OX40L”) AND (“polymorphism*” OR “variant*”) AND [“coronary artery disease” OR “CAD” OR “coronary heart disease” OR “CHD” OR “ischemic heart disease” OR “IHD” OR “angina pectoris” OR “myocardial infarction (MI)” OR “MI” OR (“coronary” AND “atherosclerosis”) OR “acute coronary syndrome”]. Moreover, the references cited by these articles were also evaluated to include additional relevant articles. Two investigators (S Liu and X Wang) independently read and checked the associated articles and retrieved articles separately.

### Eligibility Criteria

PICOS inclusion criteria were used for this meta-analysis (Supplementary Table 2). Briefly, studies were included if they met the following criteria: (1) association of *TNFSF4* polymorphisms (rs3861950 T > C and rs1234313 A > G) with susceptibility to CAD; (2) CAD diagnosis by using coronary angiography and international validated criteria; (3) case-control or cohort studies; (4) availablility of original data and sufficient genotype data provided; (5) human studies; and (6) studies subjected to the Hardy-Weinberg equilibrium (HWE), (*p* > 0.05). Additionally, the exclusion criteria were as follows: (1) duplicate studies and repetitive data; (2) reviews, meeting abstracts, and meta-analysis; (3) studies on the association between other gene polymorphisms and CAD risks; and (4) family-based and case-only studies. The χ^2^-test was used to determine whether the observed genotype frequencies conformed to HWE, and *p* < 0.05 was considered statistically significant (Thakkinstian et al., 2005; Bowden et al., 2011).

### Literature Quality Assessment

The Newcastle-Ottawa scale (NOS) was applied to assess article quality, which consists of three perspectives: selection, comparability, and exposure. A total score of five or above was considered a high-quality study, while scores lower than five were further excluded (Stang, 2010). The scores of NOS range from 0 to 9 and the scores of each study are listed in Supplementary Table 3.

### Data Extraction

The following variables for the rs3861950 T > C and rs1234313 A > G polymorphisms were extracted from the eligible studies by two investigators (S Liu and X Wang) independently: first author, publication year, type of diseases, country and ethnicity of the study population, sample size of cases and controls, genotyping methods, percentage of males and mean age of the study population, number of genotypes and alleles in both groups, and study design. All investigators participated in the discussion to reach a consensus.

### Statistical Analysis

The allele frequencies of the polymorphism from each study were calculated from genotype distributions or from the minor allele frequencies (MAF) provided in the studies. The association between the two *TNFSF4* polymorphisms and susceptibility to CAD was assessed from the odds ratios (ORs) and their corresponding 95% confidence intervals (95% CIs) for each study in five genetic models: allelic, dominant, recessive, heterozygous, and homozygous models. Complete and subgroup analyses based on ethnicity and diseases were performed. A *p* < 0.05 was considered to be statistically significant. The Cochran's Q statistical test and *I*^2^-values were used to evaluate the heterogeneity of the eligible studies, and the pooled OR and its 95% CI were obtained by using either the fixed-effect model (*p* > 0.1 and *I*^2^ ≤ 50%) or the random-effect model (*p* ≤ 0.1 or *I*^2^ > 50%)(Bowden et al., 2011). Considering the large heterogeneity, meta-regression analyses were carried out. The following variables were considered to determine the potential source: publication year, population ethnicity (Asian or Caucasian), disease type, genotype methods, source of controls, and the case sample size (defined as >500 or <500 cases);

*p* < 0.05 was considered to be statistically significant. Begg's funnel plot and the Egger's test were used to determine the publication bias with *p* < 0.05 being statistically significant (Begg and Mazumdar, 1994; Egger, 1997). The robustness of results was tested by using sensitivity analysis. STATA version 12.0 was used to perform all statistical analyses (Thakkinstian et al., 2005).

## Results

### Characteristics of the Included Studies

**Figure 1:**
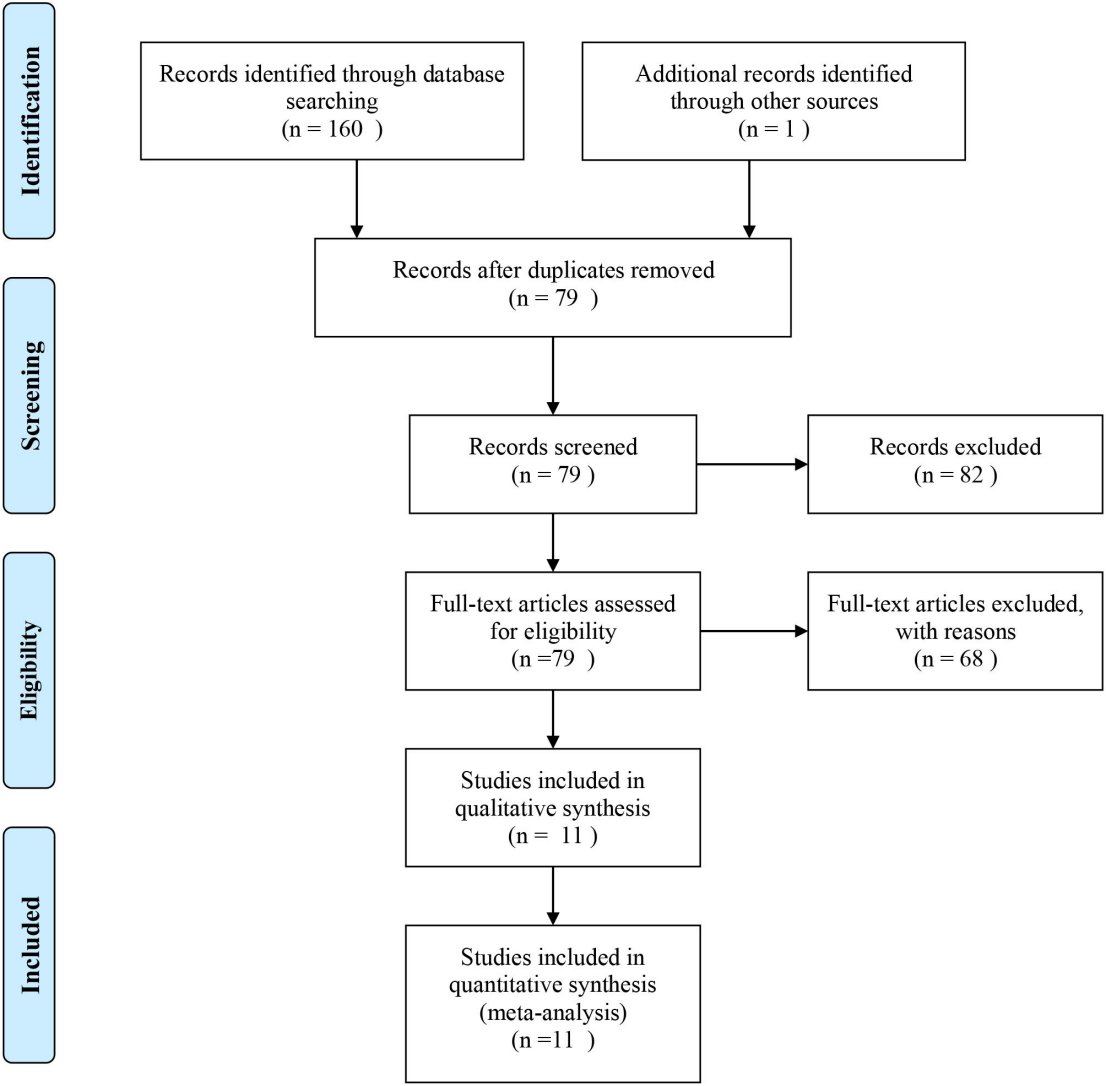
Flow diagram of the study selection process based on PRISMA guidlines.

A total of 161 papers were initially obtained from PubMed, Web of Science, EBSCO, CNKI, and Google Scholar using the keywords search before February 2020. Among these studies, 150 articles were further excluded using the selection criteria described above and 11 of the articles (Wang et al., 2005; Koch et al., 2008; Chen et al., 2011; Cheng et al., 2011, 2015; Ria et al., 2011; Feng et al., 2013; Huang et al., 2014, 2016; Jiang et al., 2019; Huang) were included for quality assessment and evaluation of the Hardy-Weinberg equilibrium (HWE) (Thakkinstian et al., 2005). Finally, the 11 eligible articles containing nine studies of rs3861950 T > C and nine studies of rs1234313 A > G were included based on NOS scores >5 (Supplementary Table 3). The overview of the study selection and exclusion process based on the PRISMA diagram is shown in Figure 1, and the main characteristics of the eligible studies are shown in Table 1.

**Table 1:** Main characteristics of the eligible studies included in this meta-analysis.

										**Case**					**Control**						
					**Sample size**					**Genetype**			**Allele**		**Genetype**			**Allele**			
**Polymorphisms**	**First author**	**Year**	**Endpoints**	**Ethnicity**	**Case**	**Control**	**Genotyping methods**	**Gender (%M) Case/control**	**Mean Age, y Case/control**	**aa**	**Aa**	**AA**	**a**	**A**	**aa**	**Aa**	**AA**	**a**	**A**	**HWE of control**	**NOS**
rs3861950	Cheng	2015	MI	Asian	285	645	PCR-LDR	77.54/58.91	62.1 ± 12.0/61.9 ± 12.0	4	67	214	75	495	18	142	485	178	1112	0.0583	7
	Huang	2015	ACVP	Asian	510	485	Taqman	38.04/26.19	61.8 ± 11.2/62.2 ± 10.7	36	179	295	251	769	14	148	396	176	940	0.969	8
	Feng	2013	CI	Asian	385	385	TaqMan	61.56/63.90	59.5 ± 11.5/60.6 ± 11.8	26	122	237	174	596	10	91	284	111	659	0.4089	7
	Ria	2011	MI	Caucasian	359	382	TaqMan	NA	NA	159	160	40	159	1959	11	157	953	179	2063	0.1171	5
	Chen	2011	CAD	Asian	498	509	PCR-RFLP	64.40/69.93	60.4 ± 10.4/60.8 ± 11.2	432	63	3	478	240	187	160	35	534	230	0.9265	7
	Cheng	2010	CAD	Asian	1,059	1,021	PCR-RFLP TaqMan	71.08/70.73	61.3 ± 10.7/60.4 ± 11.3	11	137	911	927	69	419	82	8	920	98	0.0944	7
	Koch	2008	MI	Caucasian	3,657	1,211	TaqMan	75.80/50.62	64.0 ± 12.0/60.3 ± 11.9	472	1,616	1,569	2,560	4,754	520	557	134	1,597	825	0.405	6
	Huang	2007	CI	Asian	287	285	TaqMan	65.85/57.89	59.8 ± 11.5/61.4 ± 11.9	22	65	200	109	465	6	66	213	78	492	0.7395	5
	Wang	2005	MI	Caucasian	355	373	PCR	82.30/82.40	52.0 ± 6.0/53.0 ± 5.0	157	158	40	472	238	181	158	34	520	226	0.9544	5
rs1234313	Jiang	2019	CAT	Asian	481	538	TaqMan	NA	NA	54	215	212	323	639	56	252	230	364	712	0.2835	7
	Cheng	2015	MI	Asian	285	645	PCR-LDR	38.04/26.19	61.8 ± 11.2/62.2 ± 10.7	30	126	129	186	384	91	303	251	485	805	0.9769	7
	Huang	2014	CI	Asian	450	378	TaqMan	61.60/59.00	60.4 ± 11.2/58.6 ± 10.7	51	205	194	307	593	43	169	166	255	501	0.9989	8
	Ria	2011	MI	Caucasian	359	382	TaqMan	NA	NA	31	149	179	211	507	41	169	172	251	513	0.9573	5
	Chen	2011	CAD	Asian	498	509	PCR-RFLP	64.40/69.93	60.4 ± 10.4/60.8 ± 11.2	56	213	229	325	671	68	211	230	347	671	0.0805	7
	Cheng	2010	CAD	Asian	547	601	PCR-RFLP TaqMan	71.08/70.73	61.3 ± 10.7/60.4 ± 11.3	61	240	245	362	730	79	250	272	408	794	0.0759	7
	Koch	2008	MI	Caucasian	3,657	1,211	TaqMan	75.80/50.62	64.0 ± 12.0/60.3 ± 11.9	370	1,552	1,735	2,292	5,022	117	542	552	776	1,646	0.3344	6
	Huang	2007	CI	Asian	291	303	TaqMan	65.85/57.89	59.8 ± 11.5/61.4 ± 11.9	34	134	123	202	380	39	134	130	212	394	0.6282	5
	Wang	2005	MI	Caucasian	383	392	PCR	82.30/82.40	52.0 ± 6.0/53.0 ± 5.0	34	159	190	227	539	44	174	174	262	522	0.9598	5

“a” means minor allele; “A” means major allele.

*MI, Myocardial Infarction; ACVP, Asymptomatic Carotid Vulnerable Plaque; CI, Cerebral Infarction; CAD, Coronary Artery Disease; CAT, Cerebral Arterial Thrombosis; HWE, Hardy-Weinberg equilibrium; NOS, Newcastle-Ottawa Scale; NA, not available*.

### Overall and Subgroup Meta-Analyses

Nine studies with 7,395 cases and 5,296 controls for rs3861950 T > C and nine studies with 6,951 cases and 4,959 controls for rs1234313 A > G were analyzed. The overall and subgroup meta-analyses based on ethnicity and type of disease of the association between the two *TNFSF4* SNPs and CAD risk were performed in allelic, dominant, recessive, heterozygous, and homozygous models. The results were summarized in **Table 3**.

### Association Between *TNFSF4* rs3861950 T > C Polymorphism and CAD Risk

**Figure 2:**
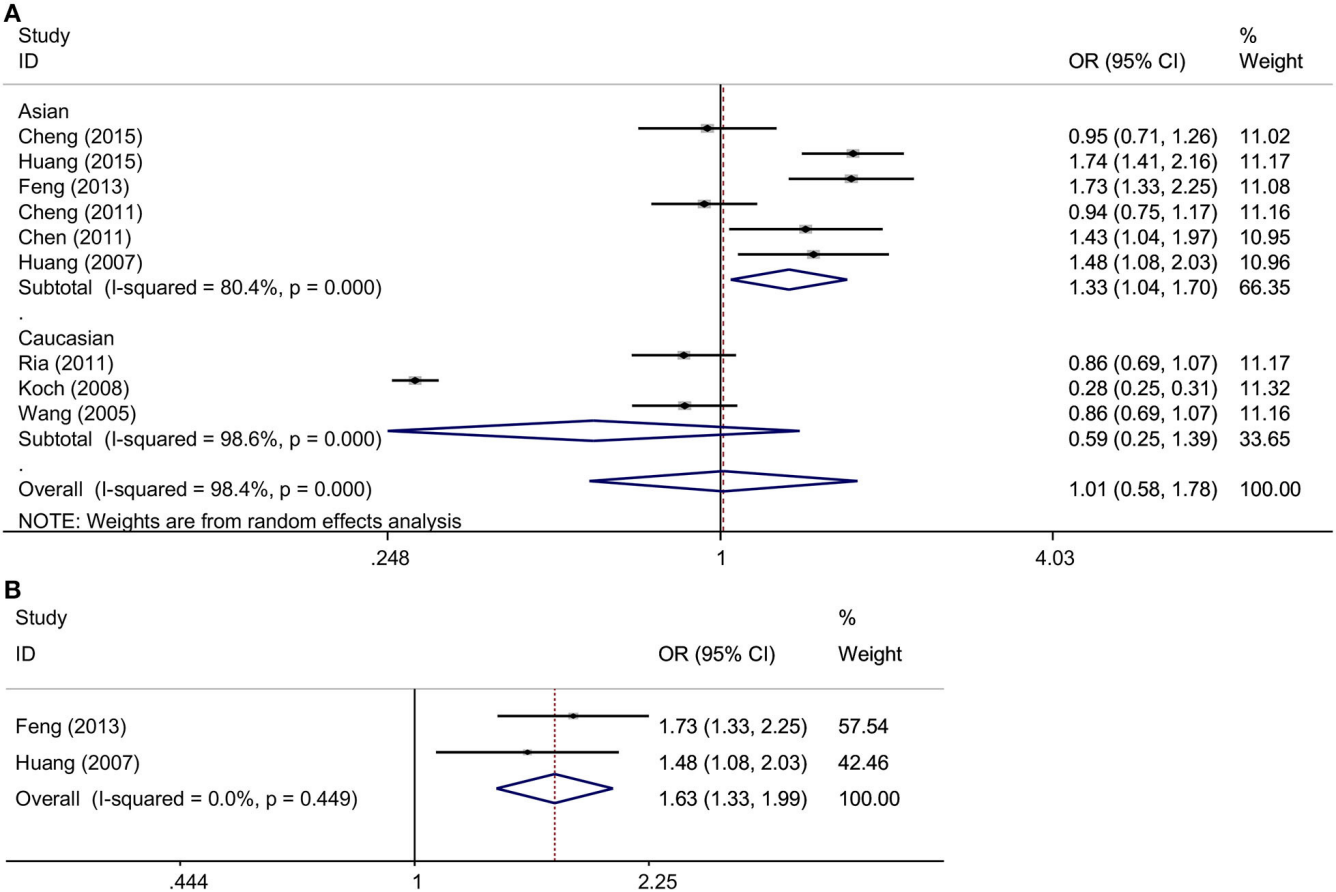
**(A)** ORs with their 95% CI for the association between *TNFSF4* rs3861950 T>C polymorphism and the susceptibility to CAD in the overall population stratified by ethnicity in the random-effects model (allelic model: C vs. T); **(B)** ORs with their 95% CI for the association between *TNFSF4* rs3861950 T>C polymorphism and the susceptibility to CI in the fixed-effects model (allelic model: C vs. T).

Six and three studies in the Asian and Caucasian populations, respectively, were included for rs3861950 T > C. Our overall analysis showed no significant correlation between the *TNFSF4* rs3861950 T > C polymorphism and CAD risk under the five genetic models (Table 2). However, we observed an increased risk of CAD risk in the Asian population in the allelic model (C vs. T, OR = 1.33, 95% CI: 1.04–1.70, *p* = 0.021), dominant model (CCCT vs. TT, OR = 1.34, 95% CI: 1.00–1.78, *p* = 0.046) and homozygous model (CC vs. TT, OR = 2.07, 95% CI: 1.12–3.81, *p* = 0.020), but not in the Caucasian population (Figure 2A and Table 2). Furthermore, subgroup analysis based on types of disease demonstrated that in *TNFSF4* rs3861950 T > C polymorphism there is a robust correlation with increased risk of cerebral infarction (CI) in the allelic model (C vs. T, OR = 1.63, 95% CI: 1.33–1.99, *p* = 0.000), dominant model (CCCT vs. TT, OR = 1.55, 95% CI: 1.22–1.96, *p* = 0.000), heterozygous model (CT vs. TT, OR = 1.35, 95% CI: 1.06–1.74, *p* = 0.017) and homozygous model (CC vs. TT, OR = 3.42, 95% CI: 1.91–6.12, *p* = 0.000) (Figure 2B and Table 2). These results indicated that *TNFSF4* rs3861950 T > C polymorphism was a risk factor for CAD, specifically in the Asian population, and this SNP increases cerebral infarction susceptibility.

**Table 2:** Overall and stratified analyses of the association between *TNFSF4* rs3861950 T > C and coronary artery disease risk.

				**Effect size**		**Heterogeneity**		
**Gene model**	**Stratify**		**Study (*n*)**	**OR (95% CI)**	***p*-value**	***I*^**2**^ (%)**	***p*-value**	**Effect model**
Allelic model (C vs. T)	Total		9	1.01 (0.58, 1.78)	0.964	98.4	0.000	R
	Disease	MI	4	0.66 (0.31, 1.39)	0.274	98.3	0.000	R
		CAD	2	1.14 (0.75, 1.72)	0.542	78.0	0.033	R
		CI	2	**1.63 (1.33, 1.99)**	**0.000**	0.0	0.449	F
	Ethnicity	Asian	6	**1.33 (1.04, 1.70)**	**0.021**	80.4	0.000	R
		Caucasian	3	0.59 (0.25, 1.39)	0.227	98.6	0.000	R
Dominant model (CCCT vs. TT)	Total		9	0.97 (0.49, 1.94)	0.934	97.6	0.000	R
	Disease	MI	4	0.57 (0.19, 1.67)	0.303	97.6	0.000	R
		CAD	2	0.96 (0.76, 1.21)	0.714	56.8	0.128	F
		CI	2	**1.55 (1.22, 1.96)**	**0.000**	38.4	0.203	F
	Ethnicity	Asian	6	**1.34 (1.00, 1.78)**	**0.046**	76.3	0.001	R
		Caucasian	3	0.46 (0.14, 1.57)	0.218	96.8	0.000	R
Recessive model (CC vs. CTTT)	Total		9	1.10 (0.53, 2.29)	0.798	95.4	0.000	R
	Disease	MI	4	1.47 (0.72, 3.01)	0.288	89.0	0.000	R
		CAD	2	0.75 (0.20, 2.80)	0.667	90.1	0.001	R
		CI	2	0.55 (0.02, 17.53)	0.736	98.8	0.000	R
	Ethnicity	Asian	6	0.92 (0.25,3.33)	0.897	96.6	0.000	R
		Caucasian	3	1.50 (0.66, 3.40)	0.337	92.6	0.000	R
Heterozygous model (CT vs. TT)	Total		9	0.96 (0.56, 1.66)	0.897	95.7	0.000	R
	Disease	MI	4	0.66 (0.28, 1.57)	0.347	95.9	0.000	R
		CAD	2	0.94 (0.74, 1.20)	0.611	23.2	0.254	F
		CI	2	**1.35 (1.06, 1.74)**	**0.017**	63.1	0.100	F
	Ethnicity	Asian	6	1.24 (0.97, 1.58)	0.086	65.1	0.014	R
		Caucasian	3	0.56 (0.21, 1.46)	0.235	94.4	0.000	R
Homozygous model (CC vs. TT)	Total		9	1.09 (0.32, 3.71)	0.891	97.5	0.000	R
	Disease	MI	4	0.38 (0.09, 1.64)	0.194	97.5	0.000	R
		CAD	2	1.41 (0.71, 2.83)	0.329	31.0	0.229	F
		CI	2	**3.42 (1.91, 6.12)**	**0.000**	0.0	0.709	F
	Ethnicity	Asian	6	**2.07 (1.12, 3.81)**	**0.020**	65.0	0.014	R
		Caucasian	3	0.35 (0.06, 1.95)	0.228	98.2	0.000	R

The statistical differences were shown in bold.

*MI, Myocardial Infarction; CAD, Coronary Artery Disease; CI, Cerebral Infarction; OR, Odds Ratio; 95% CI, 95% Confidence Interval; R, Random-effects model; F, Fixed-effects model*.

### Association Between *TNFSF4* rs1234313 A > G Polymorphism and CAD Risk

**Figure 3:**
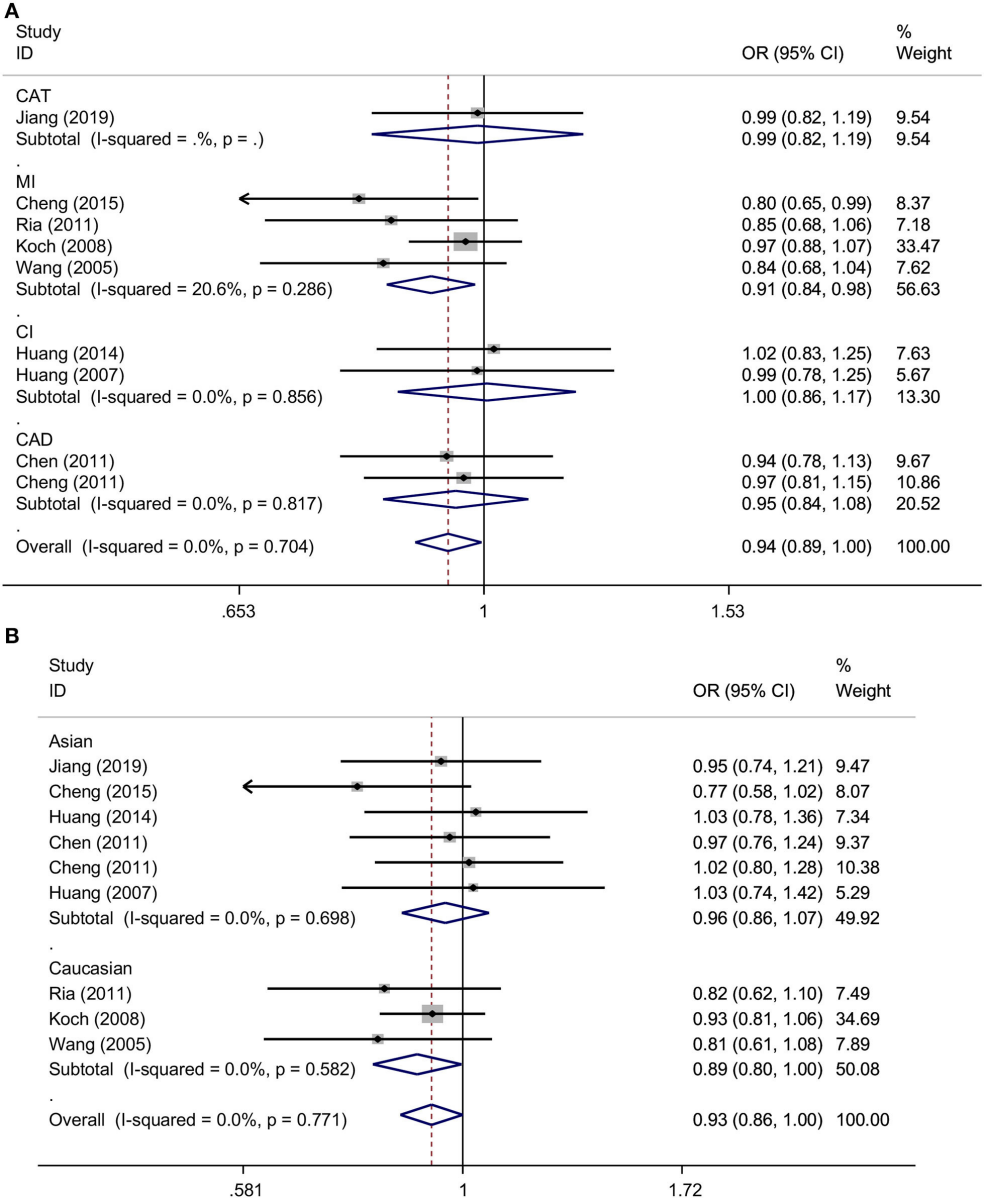
ORs with their 95% CI for the association between *TNFSF4* rs1234313 A>G polymorphism and the susceptibility to CAD in the overall population stratified by disease (allelic model: G vs. A) **(A)** and ethnicity (dominant model: GGGA vs. AA) **(B)** both in the fixed-effects model.

Overall, this meta-analysis revealed a significant association between *TNFSF4* rs1234313 A > G polymorphism and decreased risk of CAD in the allelic model (G vs. A, OR = 0.94, 95% CI: 0.89–1.00, *p* = 0.034) and dominant model (GGGA vs. AA, OR = 0.93, 95% CI: 0.86–1.00, *p* = 0.049) (Figure 3A and Table 3). Additionally, it showed that this SNP was a protective factor in the Caucasian population in the dominant model (GGGA vs. AA, OR = 0.89, 95% CI: 0.80–1.00, *p* = 0.045) and heterozygous model (GA vs. AA, OR = 0.89, 95% CI: 0.79–1.00, *p* = 0.048), but not in the Asian model (Figure 3B and Table 3). Subgroup analysis based on types of disease showed that this SNP was also a favorable marker for decreased risk of myocardial infarction (MI) in the allelic model (G vs. A, OR = 0.91, 95% CI: 0.84–0.98, *p* = 0.018), dominant model (GGGA vs. AA, OR = 0.88, 95% CI: 0.79–0.97, *p* = 0.012) and heterozygous model (GA vs. AA, OR = 0.88, 95% CI: 0.79–0.98, *p* = 0.019) (Figure 3A and Table 3). These results indicated that *TNFSF4* rs1234313 A > G polymorphism was a protective factor for CAD risk.

**Table 3:** Overall and stratified analyses of the association between *TNFSF4* rs1234313 A > G and coronary artery disease risk.

				**Effect size**		**Heterogeneity**		
**Gene model**	**Stratify**		**Study (*n*)**	**OR (95% CI)**	***p*-value**	***I*^**2**^ (%)**	***p*-value**	**Effect model**
Allelic model (G vs. A)	Total		9	**0.94 (0.89, 1.00)**	**0.034**	0.0	0.704	F
	Disease	MI	4	**0.91 (0.84, 0.98)**	**0.018**	20.6	0.286	F
		CAD	2	0.95 (0.84, 1.08)	0.443	0.0	0.817	F
		CI	2	1.00 (0.86, 1.17)	0.953	0.0	0.856	F
	Ethnicity	Asian	6	0.95 (0.88, 1.03)	0.192	0.0	0.664	F
		Caucasian	3	0.93 (0.86, 1.01)	0.087	6.9	0.342	F
Dominant model (GGGA vs. AA)	Total		9	**0.93 (0.86, 1.00)**	**0.049**	0.0	0.771	F
	Disease	MI	4	**0.88 (0.79, 0.97)**	**0.012**	0.0	0.570	F
		CAD	2	0.99 (0.84, 1.18)	0.938	0.0	0.783	F
		CI	2	1.03 (0.83, 1.27)	0.780	0.0	0.975	F
	Ethnicity	Asian	6	0.96 (0.86, 1.07)	0.434	0.0	0.771	F
		Caucasian	3	**0.89 (0.80, 1.00)**	**0.045**	0.0	0.698	F
Recessive model (GA vs. GAAA)	Total		9	0.92 (0.81, 1.04)	0.166	0.0	0.743	F
	Disease	MI	4	0.92 (0.78, 1.09)	0.329	17.7	0.303	F
		CAD	2	0.83 (0.64, 1.07)	0.150	0.0	0.966	F
		CI	2	0.95 (0.69, 1.31)	0.760	0.0	0.750	F
	Ethnicity	Asian	6	0.88 (0.75, 1.04)	0.133	0.0	0.776	F
		Caucasian	3	0.96 (0.8, 1.16)	0.689	7.0	0.341	F
Heterozygous model (GA vs. AA)	Total		9	0.94 (0.86, 1.01)	0.107	0.0	0.818	F
	Disease	MI	4	**0.88 (0.79, 0.98)**	**0.019**	0.0	0.868	F
		CAD	2	1.04 (0.87, 1.25)	0.661	0.0	0.787	F
		CI	2	1.05 (0.84, 1.31)	0.692	0.0	0.937	F
	Ethnicity	Asian	6	0.98 (0.88, 1.10)	0.756	0.0	0.758	F
		Caucasian	3	**0.89 (0.79, 1.00)**	**0.048**	0.0	0.828	F
Homozygous model (GG vs. AA)	Total		9	0.89 (0.79, 1.01)	0.075	0.0	0.699	F
	Disease	MI	4	0.86 (0.72, 1.03)	0.099	31.1	0.225	F
		CAD	2	0.84 (0.64, 1.11)	0.221	0.0	0.898	F
		CI	2	0.97 (0.69, 1.37)	0.877	0.0	0.785	F
	Ethnicity	Asian	6	0.87 (0.73, 1.04)	0.127	0.0	0.707	F
		Caucasian	3	0.91 (0.75, 1.10)	0.334	19.6	0.289	F

The statistical differences were shown in bold.

*MI, Myocardial Infarction; CAD, Coronary Artery Disease; CI, Cerebral Infarction; OR, Odds Ratio; 95% CI, 95% Confidence Interval; F, Fixed-effects model*.

### Evaluation of Heterogeneity and Sensitivity Analysis

**Figure 4:**
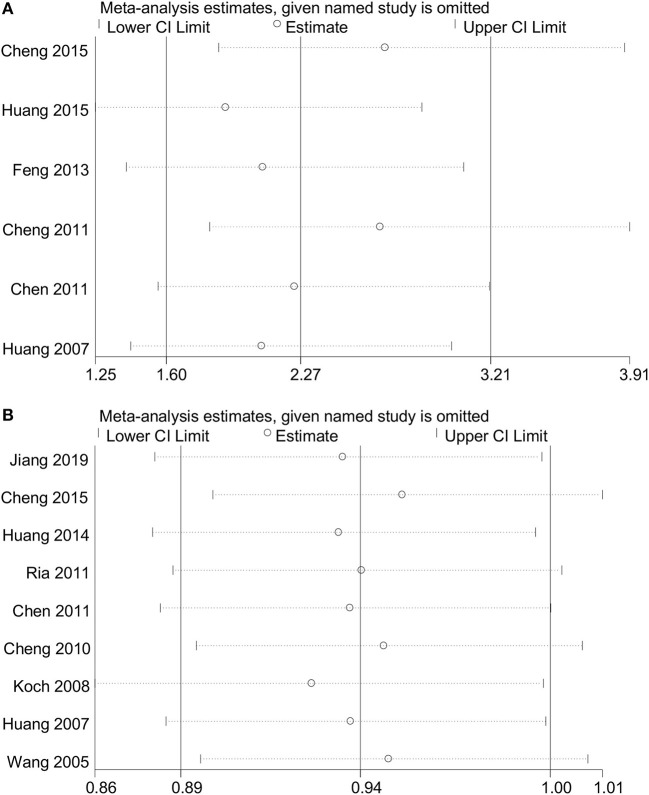
Sensitivity analyses for *TNFSF4* rs3861950 T>C **(A)** and rs1234313 A>G **(B)** polymorphisms respectively, in the Asian population (homozygous model: CC vs. TT) in random-effects model and overall (allelic model: G vs. A) population in the fixed-effects model.

No heterogeneity was observed for rs1234313 A > G polymorphism in the five genetic models analyzed in our meta-analysis (Table 3). However, although a significant association between rs3861950 T > C polymorphism and CAD risk was established under the allelic, dominant, and homozygote models in the Asian population, a large heterogeneity among studies was also observed (Table 2). Therefore, a meta-regression analysis was carried out to observe the source of heterogeneity in the general variables using data from the allelic model under the random-effects model. However, our results showed that the year of publication, population ethnicity, type of disease, genotype methods, source of controls, and the case sample size were not all associated with the large heterogeneity (*p* > 0.05). The sensitivity analysis was next conducted to determine the stability of the results. This process involved omitting one study at a time to obtain a further pooled ORs. Our results showed a significant association between rs3861950 T > C polymorphism and CAD risk remained constant in the Asian population under the homozygote model in the random-effects model (Figure 4A), which demonstrated the robustness of the results. The stability of the results was established from the sensitivity analysis of the rs1234313 A > G polymorphism in the overall population under the allelic model in the fixed-effects model (Figure 4B).

### Publication Bias

**Figure 5:**
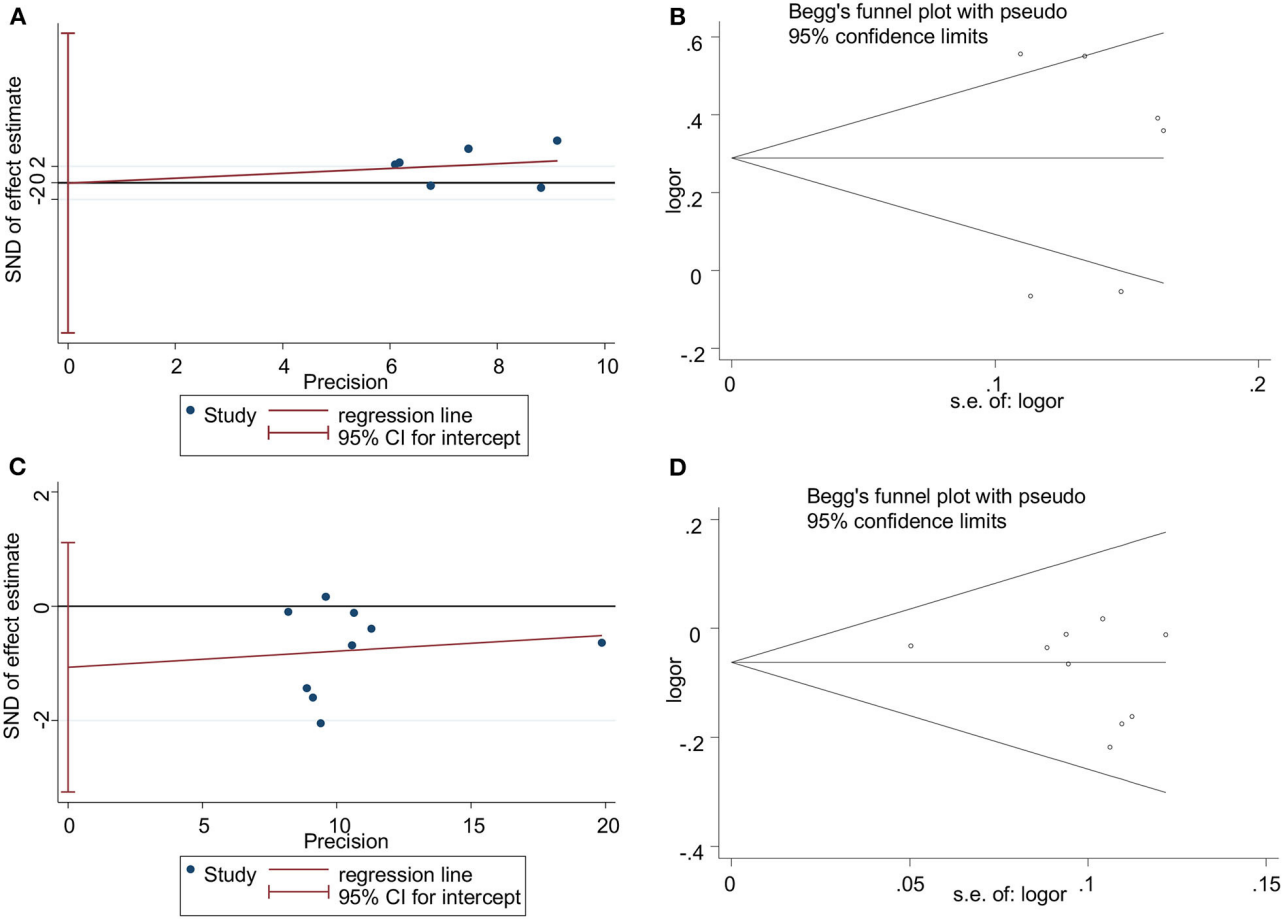
Publication bias analyzed by Egger's regression and Begg's funnel plot for *TNFSF4* rs3861950 T>C **(A,B)** and rs1234313 A>G **(C,D)** polymorphisms, respectively, in the Asian population (allelic model: C vs. T) in random-effects model and overall population (allelic model: G vs. A) in fixed-effects model.

The Egger's test and Begg's funnel plot were used to assess the publication bias of the studies involved in this meta-analysis for rs3861950 T > C and rs1234313 A > G polymorphisms (Begg and Mazumdar, 1994; Egger, 1997). The results showed that there was no statistically significant evidence of publication bias for rs3861950 T > C (Egger's test: *t* = −0.00, *p* = 0.996>0.05; Begg's test: *z* = 0.38; *p* = 0.707>0.05) (Figures 5A,B) in the Asian population and rs1234313 A > G (Egger's test: *t* = −1.16, *p* = 0.285>0.05; Begg's test: z = 0.94; *p* = 0.348>0.05; Figures 5C,D) in the overall population.

## Discussion

Because CAD is a complex metabolism-related disease, multiple separate factors should be considered for the treatment of CAD in different geographical regions. Gene sequencing studies have identified many promising biomarkers for CAD. For example, the inactivating mutant of *LDLR* (Do et al., 2015), *LPL* (Nioi et al., 2016), and *APOA5* (Do et al., 2015) has been conferred to increase the risk of CAD. In contrast, *PCSK9* (Cohen et al., 2006), *NPC1L1, APOC3* (Jørgensen et al., 2014), and *ANGPTL4* (Dewey et al., 2016) inactivating mutants have been reported to decreased CAD susceptibility. Drug therapy for specific gene mimics such as Alirocumab, Evolucumab (Cohen et al., 2006), and Ezetimibe (Nioi et al., 2016) have been approved by the Food Drug Administration (FDA) and the European Medicine Agency (EMA), but these drugs may have side effects and have shown partial toxicity in animal models (Desai et al., 2007; Dewey et al., 2016). Therefore, additional systematic analyses of the genetic function are needed to identify alternative biomarkers for diagnosis and drug development of CAD.

Previous studies revealed that TNFSF4 expression was higher in atherosclerotic lesions, and both *in vivo* and *in vitro* studies found that SIRT6 decreased *TNFSF4* gene expression by binding to and deacetylating the H3K9 at *TNFSF4* gene promoter and had a protective effect against endothelial dysfunction and atherosclerosis (Van Wanrooij et al., 2007; Xu et al., 2016), which provoked us to consider whether TNFSF4 expression and the SNPs in *TNFSF4* are closely correlated with CAD risk. *TNFSF4* rs3861950 T > C is located in the 2nd intron prior to the 3rd exon and rs1234313 A > G is located in the region of 1st intron between the 2nd and 3rd exon (Baum et al., 1994). Even though the location of the two SNPs are in the non-coding intron region, the bioinformatics analysis performed by mining the GTEx (https://gtexportal.org/home/) and HaploReg v4 (https://pubs.broadinstitute.org/mammals/haploreg/haploreg.php) databases showed that rs3861950 had little effect on *TNFSF4* expression. However, eQTL studies showed rs1234313 may have the potential to promoted the cis expression of *TNFSF4* via enhancer epigenetic regulation, but the mechanistic details need further experimental validation (Ward and Kellis, 2012). Wang et al. have assessed the associations between rs3861950 T > C and rs1234313 A > G polymorphisms with CAD risk (Wang et al., 2019). Six studies were evaluated in Wang's meta-analysis for rs3861950 T > C (Wang et al., 2005; Koch et al., 2008; Chen et al., 2011; Cheng et al., 2011, 2015; Feng et al., 2013) and another six for rs1234313 A > G (Wang et al., 2005; Koch et al., 2008; Chen et al., 2011; Cheng et al., 2011, 2015; Huang et al., 2014, 2016). However, sufficient data was only available in 4 studies, both for rs3861950 T > C (Koch et al., 2008; Chen et al., 2011; Cheng et al., 2011; Feng et al., 2013) and rs1234313 A > G (Koch et al., 2008; Chen et al., 2011; Cheng et al., 2011; Huang et al., 2014). Therefore, it was necessary to reassess their associations using a greater number of studies. The conclusions in our meta-analysis based on additional available studies revealed that rs3861950 T > C increased the risk of CAD in the Asian population under allelic, dominant, and homozygous models (Figure 2A and Table 2). In addition, analyses according to disease type showed that it could also contribute to CI risk (Figure 2B and Table 2). These results indicated that rs3861950 T>C polymorphism might be a specific risk factor in the Asian population. The genetic function of rs3861950 and the difference between Asians and other ethnicities is worth investigating further. The meta-analysis of rs1234313 A>G polymorphism in this study revealed that this SNP is significantly associated with a decreased risk of CAD. This result indicated that the rs1234313 A>G polymorphism might serve as a novel clinical biomarker for CAD diagnosis.

Furthermore, the rs1234313 A>G polymorphism was sensitive to myocardial infarction (MI) susceptibility, and ethnic subgroup analysis obtained from present data showed that this SNP might be a more promising biomarker in Caucasians than in Asians. In summary, this meta-analysis revealed that two novel SNPs in *TNFSF4* have a robust association with CAD susceptibility in different races, which may lay a foundation for precise CAD diagnosis, and provided theoretical support for personalized CAD treatment. Nevertheless, there were still some limitations that need to be addressed in this meta-analysis. First, the number of studies for each SNP in the different regions included was not abundant, and more independent studies were needed to evaluate our conclusion. The difference between ethnicities was smaller due to the limited number of studies included. Second, the number of studies for certain types of disease was not enough to perform further impartial analysis. Third, the effects of gender, age, smoking status, lifestyle, and other environmental factors on CAD were not considered in this meta-analysis due to the limitations or unavailable data. Moreover, the genotyping methods for SNPs identification were also inconsistent; all methods were based on PCR, such as PCR-RFLP, Taqman, and PCR-LDR, and each method had its own merits and limitations, so more appropriate methods and guidelines for SNPs identification needed to be discussed. Fourth, significant heterogeneity among studies on the association between rs3861950 and CAD risk in the Asian population was observed. However, the source of heterogeneity was not found in the meta-regression analysis, but the sensitivity analysis confirmed a robust association between the two. Therefore, more case-control studies with larger sample sizes and more available data are necessary to obtain a more defined conclusion in the future.

## Conclusion

Herein, this meta-analysis identified two novel gene polymorphisms of *TNFSF4*, rs3861950, and rs1234313, which may affect the predisposition to CAD in different genetic models, suggesting that these two SNPs of *TNFSF4* may potentially be used as biomarkers for CAD diagnosis.

## Data Availability Statement

The raw data supporting the conclusions of this article will be made available by the authors, without undue reservation, to any qualified researcher.

## Author Contributions

SL and XW: data curation. SL, XW, MY, and YP: formal analysis. ZX: funding acquisition. SY: methodology. GZ and ZX: supervision. MY and YP: validation. SL: writing—original draft. SY, GZ, and ZX: writing—review and editing. All authors contributed to the article and approved the submitted version.

## Conflict of Interest

The authors declare that the research was conducted in the absence of any commercial or financial relationships that could be construed as a potential conflict of interest.

## References

[B1] Baum P. R. Ramsdell F. Srinivasan S. Sorensen R. A. Watson M. L. Seldin M. F. (1994). Molecular characterization of murine and human OX40/OX40 ligand systems: identification of a human OX40 ligand as the HTLV-1-regulated protein gp34. EMBO J. 13, 3992–4001. 10.1002/j.1460-2075.1994.tb06715.x 8076595 PMC395319

[B2] Begg C. B. Mazumdar M. (1994). Operating characteristics of a rank correlation test for publication bias. Biometrics 50, 1088–1101. 10.2307/2533446 7786990

[B3] Bowden J. Tierney J. F. Copas A. J. Burdett S. (2011). Quantifying, displaying and accounting for heterogeneity in the meta-analysis of RCTs using standard and generalised Q statistics. BMC Med. Res. Methodol. 11:41. 10.1186/1471-2288-11-41 21473747 PMC3102034

[B4] Chen M. Z. Cheng G. H. Ma L. Wang H. Qiu R. F. Xue F. Z.. (2011). Association study between TNFSF4 and coronary heart disease. Yi Chuan 33, 239–245. 10.3724/SP.J.1005.2011.00239 21402531

[B5] Cheng G. Wang H. Chen M. Li L. Gong Y. Liu Q. (2011). Lack of evidence to support the association of polymorphisms within the TNFSF4 gene and coronary heart disease in a Chinese Han population. Exp. Ther. Med. 2, 275–280. 10.3892/etm.2010.188 22977497 PMC3440652

[B6] Cheng J. Cen J. M. Cai M. Y. Xu S. Li L. Li Z. C.. (2015). Association between TNFSF4 tagSNPs and myocardial infarction in a Chinese Han population. Genet. Mol. Res. 14, 6136–6145. 10.4238/2015.June.8.11 26125814

[B7] Christodoulidis G. Vittorio T. J. Fudim M. Lerakis S. Kosmas C. E. (2014). Inflammation in coronary artery disease. Cardiol. Rev. 22, 279–288. 10.1097/CRD.0000000000000006 24441047

[B8] Cohen J. C. Boerwinkle E. Mosley T. H. Hobbs H. H. (2006). Sequence variations in PCSK9, Low LDL and protection against coronary heart disease. N Engl. J. Med. 354, 1264–1272. 10.1056/NEJMoa054013 16554528

[B9] Desai U. Lee E.-C. Chung K. Gao C. Gay J. Key B.. (2007). Lipid-lowering effects of anti-angiopoietin-like 4 antibody recapitulate the lipid phenotype found in angiopoietin-like 4 knockout mice. Proc. Natl. Acad. Sci. U.S.A. 104, 11766–11771. 10.1073/pnas.0705041104 17609370 PMC1913890

[B10] Dewey F. E. Gusarova V. O'Dushlaine C. Gottesman O. Trejos J. Hunt C.. (2016). Inactivating variants in ANGPTL4 and risk of coronary artery disease. N Engl. J. Med. 374, 1123–1133. 10.1056/NEJMoa1510926 26933753 PMC4900689

[B11] Do R. Stitziel N. O. Won H. H. Jorgensen A. B. Duga S. Merlini P. A.. (2015). Exome sequencing identifies rare LDLR and APOA5 alleles conferring risk for myocardial infarction. Nature 518, 102–106. 10.1038/nature13917 25487149 PMC4319990

[B12] Egger M. (1997). Bias in meta-analysis detected by a simple, graphical test. BMJ 315, 629–634. 10.1136/bmj.315.7109.629 9310563 PMC2127453

[B13] Feng J. Liu Y. H. Yang Q. D. Zhu Z. H. Xia K. Tan X. L. (2013). TNFSF4 gene polymorphism rs3861950 but not rs3850641 is associated with the risk of cerebral infarction in a Chinese population. J. Thromb. Thrombolysis 36, 307–313. 10.1007/s11239-012-0849-9 23184501

[B14] Huang Q. Liu X. Feng J. Wen Y. Liu Y. (2016). Association between tumor necrosis factor superfamily member 4 gene polymorphism and risk of asymptomatic carotid vulnerable plaque in a Chinese population. Zhonghua Liu Xing Bing Xue Za Zhi 36, 998–1001. 26814870

[B15] Huang Q. Yang Q. D. Tan X. L. Feng J. Tang T. Xia J.. (2014). Absence of association between atherosclerotic cerebral infarction and TNFSF4/TNFRSF4 single nucleotide polymorphisms rs1234313, rs1234314 and rs17568 in a Chinese population. J. Int. Med. Res. 42, 436–443. 10.1177/0300060514521154 24595151

[B16] Huang Q The study on the association between TNFSF4/TNFRSF4 gene and cerebral infarction (doctoral dissertation), Central South University, Changsha, China.

[B17] Jiang Y. Liu X. Du Y. Zhou S. (2019). rs1234313 and rs45454293 are risk factors of cerebral arterial thrombosis, large artery atherosclerosis, and carotid plaque in the Han Chinese population: a case-control study. BMC Neurol. 19:31. 10.1186/s12883-019-1259-9 30797237 PMC6387510

[B18] Jørgensen A. B. Frikke-Schmidt R. Nordestgaard B. G. Tybjærg-Hansen A. (2014). Loss-of-function mutations in APOC3 and risk of ischemic vascular disease. J. Vasc. Surg. 371:1096. 10.1016/j.jvs.2014.08.087 24941082

[B19] Kaur D. Brightling C. (2012). OX40/OX40 ligand interactions in T-cell regulation and asthma. Chest 141, 494–499. 10.1378/chest.11-1730 22315115 PMC3277294

[B20] Kessler T. Vilne B. Schunkert H. (2016). The impact of genome-wide association studies on the pathophysiology and therapy of cardiovascular disease. EMBO Mol. Med. 8, 688–701. 10.15252/emmm.201506174 27189168 PMC4931285

[B21] Khera A. V. Kathiresan S. (2017). Genetics of coronary artery disease: discovery, biology and clinical translation. Nat. Rev. Genet. 18, 331–344. 10.1038/nrg.2016.160 28286336 PMC5935119

[B22] Koch W. Hoppmann P. Mueller J. C. Schomig A. Kastrati A. (2008). Lack of support for association between common variation in TNFSF4 and myocardial infarction in a German population. Nat. Genet. 40, 1386–1387. 10.1038/ng1208-1386 19029970

[B23] Marenberg M. E. Risch N. Berkman L. F. Floderus B. de Faire U. (1995). Genetic susceptibility to death from coronary heart disease in a study of twins. J. Occupat. Environ. Med. 330, 1041–1046. 10.1056/NEJM199404143301503 8127331

[B24] Moher D. Liberati A. Tetzlaff J. Altman D. G. (2009). Preferred reporting items for systematic reviews and meta-analyses: the PRISMA statement. PLoS Med. 6:e1000097 10.1371/journal.pmed.1000097 19621072 PMC2707599

[B25] Nioi P. Sigurdsson A. Thorleifsson G. Helgason H. Stefansson K. (2016). Variant ASGR1 associated with a reduced risk of coronary artery disease. N Engl. J. Med. 374:2131. 10.1056/NEJMoa1508419 27192541

[B26] Ria M. Lagercrantz J. Samnegård A. Boquist S. Hamsten A. Eriksson P. (2011). A common polymorphism in the promoter region of the TNFSF4 gene is associated with lower allele-specific expression and risk of myocardial infarction. PLoS ONE 6:e17652. 10.1371/journal.pone.0017652 21445270 PMC3060868

[B27] Stang A. (2010). Critical evaluation of the Newcastle-Ottawa scale for the assessment of the quality of nonrandomized studies in meta-analyses. Eur. J. Epidemiol. 25, 603–605. 10.1007/s10654-010-9491-z 20652370

[B28] Thakkinstian A. McElduff P. D'Este C. Duffy D. Attia J. (2005). A method for meta-analysis of molecular association studies. Stat. Med. 24, 1291–1306. 10.1002/sim.2010 15568190

[B29] Van Wanrooij E. J. A. van Puijvelde G. H. M. de Vos P. Yagita H. va Berkel T. J. C. Kuiper J. (2007). Interruption of the Tnfrsf4/Tnfsf4 (OX40/OX40L) pathway attenuates atherogenesis in low-density lipoprotein receptor-deficient mice. Arterioscler. Thromb. Vasc. Biol. 27, 204–210. 10.1161/01.ATV.0000251007.07648.81 17068285

[B30] Wang X. Luan Y. Zhang C. (2019). A meta-analysis on correlations of OX40L variants with atherosclerotic disorders. J. Cell. Biochem. 120, 9624–9630. 10.1002/jcb.28240 30614039

[B31] Wang X. Ria M. Kelmenson P. M. Eriksson P. Higgins D. C. Samnegård A.. (2005). Positional identification of TNFSF4, encoding OX40 ligand, as a gene that influences atherosclerosis susceptibility. Nat. Genet. 37, 365–372. 10.1038/ng1524 15750594

[B32] Ward L. D. Kellis M. (2012). HaploReg: a resource for exploring chromatin states, conservation, and regulatory motif alterations within sets of genetically linked variants. Nucleic Acids Res. 40, D930–D934. 10.1093/nar/gkr917 22064851 PMC3245002

[B33] Xu S. Yin M. Koroleva M. Mastrangelo M. A. Zhang W. Bai P.. (2016). SIRT6 protects against endothelial dysfunction and atherosclerosis in mice. Aging 8, 1064–1082. 10.18632/aging.100975 27249230 PMC4931854

