# Supplementary material for: A Meta-Analysis on the Association Between TNFSF4 Polymorphisms (rs3861950 T > C and rs1234313 A > G) and Susceptibility to Coronary Artery Disease
Source: Front Physiol. 2020 Nov 26;11:539288. doi: 10.3389/fphys.2020.539288 (PMC7732687; doi:10.3389/fphys.2020.539288)
Supplement: Supplementary file 2 [file Table_2.docx]

Table S2 The literature search based on the PICOS framework

| Participant | Intervention | Comparator | Outcome | Study design | Limits |
| --- | --- | --- | --- | --- | --- |
| Hospital population;  Community poplation | (tumor necrosis factor superfamily member 4" OR "TNFSF4" OR "OX40L") AND ("polymorphism∗" OR "variant∗") | No limitation | No limitation | Case-control study；  cohort study | No languge limitation；  Human trials |
