# Supplementary material for: A Meta-Analysis on the Association Between TNFSF4 Polymorphisms (rs3861950 T > C and rs1234313 A > G) and Susceptibility to Coronary Artery Disease
Source: Front Physiol. 2020 Nov 26;11:539288. doi: 10.3389/fphys.2020.539288 (PMC7732687; doi:10.3389/fphys.2020.539288)
Supplement: Supplementary file 3 [file Table_3.docx]

Table S3. NOS-based quality assessment of the eligible studies.

| Study | Selection | | | | Comparability | Exposure | | | Total |
| --- | --- | --- | --- | --- | --- | --- | --- | --- | --- |
|  | Definition | Representativeness | Selection | Definition |  | Ascertainment | Method | Rate |  |
| Cheng 2015 | 1 | 1 | 0 | 1 | 2 | 1 | 1 | 0 | 7 |
| Huang 2015 | 1 | 1 | 1 | 1 | 2 | 1 | 1 | 0 | 8 |
| Feng 2013 | 1 | 1 | 0 | 1 | 2 | 1 | 1 | 0 | 7 |
| Ria 2011 | 1 | 1 | 0 | 0 | 1 | 1 | 1 | 0 | 5 |
| Chen 2011 | 1 | 1 | 0 | 1 | 2 | 1 | 1 | 0 | 7 |
| Cheng 2010 | 1 | 1 | 0 | 1 | 2 | 1 | 1 | 0 | 7 |
| Koch 2008 | 1 | 1 | 0 | 1 | 1 | 1 | 1 | 0 | 6 |
| Huang 2007 | 1 | 1 | 0 | 1 | 0 | 1 | 1 | 0 | 5 |
| Wang 2005 | 1 | 1 | 0 | 0 | 1 | 1 | 1 | 0 | 5 |
| Jiang 2019 | 1 | 1 | 0 | 1 | 2 | 1 | 1 | 0 | 7 |
| Huang 2014 | 1 | 1 | 1 | 1 | 2 | 1 | 1 | 0 | 8 |
|  |  |  |  |  |  |  |  |  |  |
